# RETRACTED ARTICLE: Preliminary proposal: a classification system for reconstruction with autologous femoral head after periacetabular tumors resection

**DOI:** 10.1186/s13018-021-02275-y

**Published:** 2021-02-08

**Authors:** Chunzhi Yi, Jiaqian Zheng, Ruoyu Li, Yun Lan, Mincong He, Jieqing Lai, Tianan Guan, Fengxiang Pang, Zongquan Mo, Peng Chen, Yue Li, Nannan Zhou, Xingfu Yang, Bin Fang

**Affiliations:** 1 Department of Orthopedic Oncology, The First Affiliated Hospital of Guangzhou University of Chinese Medicine, Guangzhou University of Chinese Medicine, Guangzhou, PR China; 2 The First Clinical Medical College, Guangzhou University of Chinese Medicine, Guangzhou, PR China

The Editor-in-Chief has retracted this article because the authors did not obtain informed consent to publish identifying information about the patients involved in the study. The content of this article is no longer available online to protect patient confidentiality. Zheng Jiaqian agrees to this retraction. Fang Bin agrees with the the retraction but disagrees with the retraction wording. All other authors did not respond to correspondence from the publisher about this retraction.

